# Hypertension and frailty in older adults: a bibliometric analysis and knowledge mapping based on Web of Science, Scopus, and PubMed (1973–2025)

**DOI:** 10.3389/fmed.2026.1818161

**Published:** 2026-06-18

**Authors:** Shiyang Yu, Wen Yang, Rui Luo, Jinyi Li, Min Liu

**Affiliations:** 1 Geriatric Department, The First People's Hospital of Longquanyi District, Chengdu, Sichuan, China; 2 Urology Department, The First People's Hospital of Longquanyi District, Chengdu, Sichuan, China; 3 General Medicine Department, The First People's Hospital of Longquanyi District, Chengdu, Sichuan, China; 4 Cardiovascular Medicine Department, The First People's Hospital of Longquanyi District, Chengdu, Sichuan, China

**Keywords:** bibliometrics, body composition, citation analysis, exercise intervention, frailty, frailty index, gait speed, grip strength

## Abstract

**Background:**

Hypertension and frailty frequently coexist in older adults and jointly increase the risks of falls, hospitalization, disability, and mortality. However, bibliometric evidence mapping the knowledge structure, hotspots, and trends in this field remains limited. This study aimed to characterize the intellectual landscape of hypertension and frailty research using a multi-database bibliometric approach.

**Methods:**

English-language publications were retrieved from the Web of Science Core Collection (WoSCC), Scopus, and PubMed. Article and Review types were restricted in WoSCC and Scopus, while PubMed records were harmonized during preprocessing. Records were merged in R using bibliometrix and deduplicated mainly by DOI and title. Annual trends, countries/regions, institutions, authors, journals, and collaboration patterns were analyzed. VOSviewer and bibliometrix were used for keyword co-occurrence and institutional collaboration networks. CiteSpace, based on WoSCC data, was used for keyword clustering, citation bursts, and citation analyses.

**Results:**

In total, 1,853 records were retrieved from WoSCC, 5,210 from Scopus, and 1,615 from PubMed; 4,954 publications were included after cleaning. Publications were sparse from 1973 to 2000, increased after 2000, accelerated after 2010, and rose sharply after 2015, approaching 1,000 in 2023. The United States contributed the most publications (1,086; 21.9%), followed by China (542; 10.9%) and Italy (379; 7.7%). Wang Y was the most productive author, Archives of Gerontology and Geriatrics was the leading journal, and Harvard University was the most productive institution. Keyword analyses highlighted hypertension management, frailty indices, body composition, grip strength, gait speed, physical activity, and successful aging. CiteSpace identified recent citation bursts for “frailty index” and “hypertension management.” The PURE study ranked first in global citations, while the SPRINT trial in adults aged ≥75 years ranked first in local citations.

**Conclusions:**

Research on hypertension and frailty has grown rapidly, especially after 2015, forming a knowledge structure focused on hypertension management, frailty assessment, and functional outcomes. Future studies should address multimorbid populations, prioritize functional endpoints, and validate combined blood pressure control and function-oriented interventions in real-world settings.

## 1 Introduction

With the accelerating pace of global population aging, chronic non-communicable diseases and geriatric syndromes are posing increasingly severe challenges to public health systems. Hypertension, one of the most prevalent chronic non-communicable diseases worldwide, is a leading risk factor for the incidence and mortality of major conditions such as cardiovascular disease, stroke, and chronic kidney disease (1, 2). It is estimated that approximately one billion people are affected by hypertension globally (3). Frailty is a geriatric syndrome characterized by diminished physiological reserves, multisystem functional decline, and reduced tolerance to stressors (4). It typically manifests as reduced physical capacity, fatigue, impaired mobility, and an overall decline in quality of life, reflecting both physical and psychological vulnerability. As aging progresses, frailty represents a sustained deterioration in the body's ability to maintain homeostasis and has been widely recognized as a strong predictor of adverse health outcomes, including falls, hospitalization, disability, and death (5–7). Previous studies have shown that frailty is closely associated with multiple factors, including advanced age, malnutrition, low physical activity, cardiovascular disease, and psychosocial determinants (8, 9). A meta-analysis by Li et al. (10) on community-dwelling older adults demonstrated that frail individuals had a significantly higher risk of all-cause mortality than their robust counterparts, highlighting frailty as an emerging priority in geriatrics and public health research.

In recent years, research on the relationship between hypertension and frailty has increased steadily. Hypertension not only contributes to a broad range of complications but may also elevate the risk of developing frailty-related conditions. The accumulation of subclinical disorders and comorbidities can accelerate the decline of physiological reserves across multiple systems, disrupt age-related homeostatic balance, and ultimately facilitate the onset of frailty (11). Mechanistically, hypertension may promote arterial stiffness, endothelial dysfunction, and impaired baroreflex sensitivity, thereby increasing the risk of vascular events and potentially compromising functional reserve. With progressive vascular aging, reduced tissue perfusion may further exacerbate functional deterioration and accelerate frailty development. Once frailty occurs, it may in turn aggravate blood pressure instability and reduce tolerance to antihypertensive medications, creating a vicious cycle. The interplay between hypertension and frailty is underpinned by complex biological mechanisms, involving chronic low-grade inflammation, mitochondrial dysfunction, endocrine dysregulation, and impaired repair processes. These physiological changes not only contribute to ongoing declines in functional reserve but also promote multimorbidity and increase the overall burden of chronic disease. Frailty is closely linked to the coexistence of multiple chronic conditions and may form disease clusters—such as cardiometabolic, inflammation–musculoskeletal, and neurovascular–cognitive phenotypes—that share overlapping biological pathways; these interactions can further worsen prognosis as aging and multimorbidity progress (12).

Although numerous original studies and systematic reviews have examined the clinical characteristics and health impacts of hypertension or frailty, bibliometric research that systematically delineates the developmental trajectory, knowledge structure, and research hotspots of the hypertension–frailty interface remains relatively limited. In particular, comprehensive assessments of global publication trends, leading countries and institutions, major author collaboration networks, thematic evolution, and emerging frontiers are still lacking, which may hinder evidence integration and the precise identification of priorities for future research. Therefore, this study employs bibliometric methods to systematically retrieve publications on the relationship between hypertension and frailty from WoSCC, Scopus, and PubMed, and to comprehensively analyze publication trends, geographical and institutional distributions, core journals and highly cited papers, keyword co-occurrence, and thematic evolution. By mapping the knowledge landscape and developmental patterns of this field, we aim to identify research hotspots and potential gaps, thereby providing scientific evidence to inform subsequent mechanistic studies and the development of clinical intervention strategies.

## 2 Methods

### 2.1 Data sources and search strategy

We systematically searched three databases—WoSCC, Scopus, and PubMed—to comprehensively capture international literature in this field. To enhance comparability across databases, we included only English-language publications. PubMed was restricted to English at the search stage, and WoSCC and Scopus were likewise limited to English records. Regarding document types, WoSCC and Scopus were restricted to Article and Review during retrieval. Because publication-type indexing in PubMed may be inconsistent, no document-type restriction was applied in PubMed at the search stage; instead, records were standardized during subsequent preprocessing.

Database-specific search queries were developed according to each platform's indexing rules while maintaining consistent core concepts—hypertension and frailty. In WoSCC, the Topic field (TS) was used: TS = (“hypertension” OR “high blood pressure” OR hypertensive OR hyperpiesis OR hypertonia OR “essential hypertension” OR “primary hypertension” OR “secondary hypertension” OR “resistant hypertension” OR “renal hypertension” OR “renovascular hypertension”) AND TS = (frail^*^) (13, 14). In Scopus, the TITLE-ABS-KEY field was used: TITLE-ABS-KEY (“hypertension” OR “high blood pressure” OR hypertensive OR hyperpiesis OR hypertonia OR “essential hypertension” OR “primary hypertension” OR “secondary hypertension” OR “resistant hypertension” OR “renal hypertension” OR “renovascular hypertension”) AND TITLE-ABS-KEY (frail^*^) (15–17). In PubMed, Medical Subject Headings (MeSH) and free-text terms (Title/Abstract) were combined to improve sensitivity and specificity: (“Hypertension” [Mesh] OR “Blood Pressure, High” [Mesh] OR hypertension [Title/Abstract] OR “high blood pressure” [Title/Abstract] OR hypertensive [Title/Abstract] OR “essential hypertension” [Title/Abstract] OR “primary hypertension” [Title/Abstract] OR “secondary hypertension” [Title/Abstract] OR “resistant hypertension” [Title/Abstract] OR “renovascular hypertension” [Title/Abstract]) AND (“Frailty” [Mesh] OR frailty [Title/Abstract] OR frail^*^[Title/Abstract]). Overall, these strategies were constructed around the same research topic and adapted to each database's indexing characteristics to ensure comprehensive, systematic, and comparable retrieval. To further assess the robustness of the search strategy, we conducted a supplementary sensitivity search. Based on the main search strategy, additional frailty-related alternative keywords with broader conceptual boundaries were incorporated, including “sarcopenia,” “muscle weakness,” “functional decline,” “physical function decline,” and “disability.” The expanded search results were then compared with the main search results to evaluate whether the alternative keywords substantially broadened the retrieval scope and introduced topic contamination. The sensitivity search was performed solely to validate the robustness of the main search strategy and was not included in the final bibliometric analysis dataset. The relevant results are presented in Supplementary Table S1.

### 2.2 Data export and integration

After the search was completed, records from each database were exported separately. Records from WoSCC were exported in plain text format as full records and cited references; records from Scopus were exported in BibTeX format; and records from PubMed were exported in Citation Manager (.nbib) format. All exported records were imported into the R environment (version 4.5.2) for processing. R is an open-source programming language and statistical computing environment that is widely used for data cleaning, statistical analysis, and visualization.

In this study, R was used to convert formats, organize fields, merge records, remove duplicates, and export bibliographic records from different databases, thereby ensuring the consistency and traceability of the multi-database integration process. Specifically, the convert2df() function in the bibliometrix package was used to convert the original export files from WoSCC, Scopus, and PubMed into a unified data frame format (18). Subsequently, the bind_rows() function in the dplyr package was used to merge records from the three databases into a comprehensive dataset. During the initial merging stage, duplicate records across different databases were retained to avoid the premature removal of potentially useful information. The final cleaned dataset was exported as an Excel file using the openxlsx package for subsequent verification and result organization.

In the R processing workflow, records from WoSCC, Scopus, and PubMed were first converted into separate data frames and then merged into an overall data frame. Key fields, including DOI, title, and document type, were then standardized, and the final analytical dataset was generated. The main R steps used in this process included convert2df() for format conversion, bind_rows() for merging the three databases, mutate() for field standardization, distinct() for duplicate removal, filter() for document type screening, and write.xlsx() for exporting the cleaned dataset.

During data integration, core fields were harmonized and standardized, including authors, title, journal name, publication year, DOI, abstract, keywords, institutional information, and document type. Because WoSCC, Scopus, and PubMed differ in their indexing systems and field structures, we retained the original fields available from each database and mapped comparable fields to unified variables. For example, WoSCC and Scopus generally provide author keywords and indexed keywords, whereas PubMed relies more heavily on MeSH terms and the Publication Type field. WoSCC provides complete cited reference information, whereas PubMed generally does not include complete reference lists. For fields that were missing in one database but available in another, no manual imputation was performed to avoid introducing additional bias.

It should be noted that although WoSCC provides citation and cited reference information, WoSCC records were first integrated with Scopus and PubMed records during the multi-database integration process to construct a comprehensive bibliographic dataset. The merged WoSCC, Scopus, and PubMed dataset was mainly used for analyses of annual publication trends, countries/regions, institutions, authors, journals, and keyword-based thematic structures. In contrast, analyses involving co-citation networks, citation relationships, and citation burst detection were conducted only using WoSCC data, which contained complete cited reference fields.

### 2.3 Deduplication and document-type cleaning

To address duplicate records that may arise after merging multiple databases, the merged dataset was uniformly cleaned and deduplicated in the R environment. First, the DOI and title fields were standardized, including harmonizing letter case, removing extra spaces, and standardizing DOI formats as much as possible. Duplicate removal was then performed based on the standardized DOI and title information to reduce the influence of the same publication appearing repeatedly across WoSCC, Scopus, and PubMed. In R, this process was mainly implemented using mutate() to standardize the DOI and title fields and distinct() to retain unique records.

For records with missing DOI information, inconsistent DOI records, or differences in title formatting, additional manual verification was conducted by combining title, publication year, and source information. This was done to ensure that each publication was retained only once while minimizing the risk of incorrectly merging different publications.

Because the indexing of publication types in PubMed is not completely consistent with that in WoSCC and Scopus, PubMed records were standardized for document type during the preprocessing stage. Specifically, the publication type field in PubMed records was first extracted. Records containing “Journal Article” were classified as Article, whereas records containing “Review,” “Systematic Review,” or “Meta-Analysis” were classified as Review. Non-research or non-review publication types, such as Editorial, Letter, Comment, News, Correction, Erratum, Case Reports, and Published Erratum, were excluded. For records containing multiple PubMed publication type labels, classification was primarily based on whether the record belonged to Article or Review. In unclear cases, manual verification was performed by jointly considering the title, abstract, and publication type information.

After cross-database deduplication was completed, the document types of records from WoSCC, Scopus, and PubMed were further harmonized, and only Articles and Reviews were retained. Non-target document types, including conference abstracts, books, editorials, letters, news items, comments, and errata, were excluded. These screening steps were mainly implemented during the data preprocessing stage, with the aim of maximizing the coverage of the initial search while ensuring consistency in document types within the final analytical dataset. The detailed screening process is shown in Supplementary Figure S1.

To evaluate the consistency of the cross-database deduplication process, we recorded the initial number of records retrieved from each database, the number of records after merging, the number of records after deduplication, and the final number of publications included in the analysis. The relevant results are presented in Supplementary Figure S1 and Supplementary Table S2. This deduplication workflow helped reduce missed duplicate records caused by relying solely on DOI matching, while also lowering the risk of residual duplicate records due to differences in title formatting.

### 2.4 Bibliometric analyses

Based on the final cleaned dataset, we applied bibliometric methods to systematically describe the overall developmental trends and structural characteristics of research on hypertension and frailty. The analyses included annual publication trends, which were used to describe changes in research output over time; country/region productivity, which was used to evaluate research output by geographic location, with this module only counting the number of publications and not including citation-based indicators; institutional productivity and collaboration networks, which were used to identify highly productive institutions and construct collaboration networks based on institutional co-occurrence; author productivity, which was used to identify highly productive authors; and journal distribution, which was used to determine the main source journals and their publication patterns.

In the analysis of institutional contributions, in addition to reporting the raw number of publications, we further calculated the proportion of publications contributed by each institution relative to the total number of publications included in the final dataset, in order to improve comparability across institutions. For institutional names with different spellings, abbreviations, or variations in affiliation systems, name standardization was performed. For example, different forms referring to the same university system, affiliated hospital, or research center were merged or uniformly labeled after manual verification. The institutional collaboration network was constructed based on institutional co-occurrence relationships and was mainly used to illustrate collaboration structures, rather than to directly evaluate the academic quality of institutions.

### 2.5 Keyword co-occurrence analysis tools

We used three tools—CiteSpace, VOSviewer, and bibliometrix—for keyword co-occurrence and bibliometric analyses. VOSviewer was applied to the merged dataset derived from WoSCC, Scopus, and PubMed. Bibliometrix was mainly used for data conversion, merging, basic bibliometric statistics, and partial network analysis, rather than as a tool for validating keyword clustering results (18). CiteSpace analysis was conducted using WoSCC data only.

CiteSpace is a visualization tool for bibliometrics and knowledge mapping that can be used to explore temporal-spatial patterns, scholarly networks, and emerging trends (19). In this study, CiteSpace was used for citation burst detection and network visualization to identify keywords, references, and topics with strong citation bursts and to track changes in research hotspots. The CiteSpace parameters were set as follows: the data source was WoSCC; node types included documents, authors, and source journals, which were selected according to the specific analysis; the analytical method included citation burst detection; the time span was set to 2015–2025; the time slice was set to 1 year; and cosine similarity was used for link strength. The 2015–2025 period was selected as the main analytical time window to focus on the rapid development stage of research at the intersection of hypertension and frailty in recent years and to improve the sensitivity for identifying emerging research hotspots. A 1-year time slice was used to capture annual changes in burst topics in greater detail.

To evaluate the stability of citation burst detection results under different time-slicing parameters, we conducted supplementary parameter testing. In addition to the 1-year time slice used in the main analysis, 2-year and 3-year time-slice settings were also tested, and the consistency of the main burst themes was compared to assess the robustness of the CiteSpace results. The relevant results are presented in Supplementary Table S3.

VOSviewer was used to construct the keyword co-occurrence network and institutional collaboration network (20). In the keyword co-occurrence analysis, the analysis type was set to co-occurrence, the unit of analysis was set to all keywords, the counting method was set to full counting, and no additional VOSviewer thesaurus file was used. The minimum keyword occurrence threshold was set to 10; among all 5,411 keywords, 251 met this threshold and were included in the visualization analysis. The choice of this threshold was mainly based on parameter testing and map interpretability. Specifically, when the threshold was set to five, more keywords were included and the thematic coverage was broader, but the network structure was more complex, noise from low-frequency keywords increased, and the boundaries between themes were less clear. When the threshold was set to 15, the network structure became more simplified, but some medium-frequency keywords with thematic relevance might have been excluded, thereby reducing thematic coverage. In contrast, when the threshold was set to 10, the number of keywords, network density, and clarity of thematic clustering were relatively balanced, allowing the main research themes to be retained while improving the readability and interpretability of the visualization. Therefore, this study ultimately adopted a minimum keyword occurrence threshold of 10 as the main analytical parameter. The stability analysis results under different keyword occurrence thresholds are presented in Supplementary Table S4.

The institutional collaboration network was also constructed using VOSviewer. The analysis type was set to co-authorship, the unit of analysis was set to organizations, and the counting method was set to full counting. To reduce the influence of large multicenter studies on the density of the collaboration network, records with more than 25 co-authoring institutions per publication were ignored. The institutional inclusion threshold was set at a minimum of 10 publications, and the minimum citation threshold was set to 0. Among all 3,118 institutions, 88 met the threshold and were included in the institutional collaboration network analysis. These parameter settings were intended to retain the main research themes and core collaborating institutions while reducing network noise from low-frequency nodes, thereby improving the readability and interpretability of the maps.

### 2.6 Citation relationship analyses

#### 2.6.1 Data source and analytical scope

Because citation relationship analyses (e.g., co-citation analysis and citation indicators) require complete reference lists and PubMed does not provide citation data, all citation-based analyses in this study were conducted using WoSCC records only, which contain complete cited-reference information. This approach helped avoid systematic bias caused by differences across databases in citation coverage, reference formats, and citation-counting rules.

#### 2.6.2 Analytical procedures

From WoSCC records, we extracted citation counts and cited references, and conducted:

Document co-citation analysis, constructing a co-citation network based on pairs of references co-cited by subsequent publications to reveal the knowledge base and classic literature structure;

Author co-citation analysis, identifying influential author groups based on author-level co-citation patterns; Source (journal) co-citation analysis, identifying core journals and their structural positions in scholarly communication.

#### 2.6.3 Interpretation and methodological consistency

It should be noted that citation relationship analyses reflect a publication's relative influence and structural position within scholarly networks and should not be directly interpreted as quality appraisal. Moreover, because databases differ in citation coverage and counting rules, we did not incorporate PubMed or Scopus data into citation relationship analyses to avoid systematic bias due to incomplete or heterogeneous citation information. Therefore, a modular strategy was adopted: the merged multi-database dataset (WoSCC, Scopus, and PubMed) was used to describe research output and thematic structures, whereas citation-based analyses (co-citation networks and citation indicators) were conducted using WoSCC data only. This approach balances breadth of coverage and methodological reliability (21). This approach helped avoid systematic bias caused by differences across databases in citation coverage, reference formats, and citation-counting rules.

### 2.7 Robustness and sensitivity analyses

To address potential retrieval bias, deduplication bias, and parameter-selection bias in multi-database bibliometric research, we further conducted several robustness and sensitivity analyses. First, the main search strategy was validated through a sensitivity analysis by adding alternative frailty-related keywords, in order to assess the stability of the search results and determine whether overly broad keywords might introduce topic contamination. Second, the consistency of the cross-database deduplication process was evaluated by recording the initial number of records retrieved from each database, the number of records after merging, the number of records after deduplication, and the final number of publications included in the analysis. Third, the time-slicing parameters in CiteSpace were modified to compare changes in citation burst detection results and assess the stability of burst theme identification. Fourth, the minimum keyword occurrence threshold in VOSviewer was adjusted to compare changes in the number of keyword nodes, network density, and main thematic directions under different thresholds, thereby evaluating the robustness of keyword co-occurrence results to parameter settings. The results of these analyses are presented in Supplementary Tables S1–S4, and the rationale for the keyword threshold selection has been added to the Methods section.

## 3 Results

### 3.1 Study selection and data synthesis

In this study, we retrieved publications related to “hypertension” and “frailty” from three databases: WoSCC, Scopus, and PubMed. Specifically, 1,853 records were identified from Web of Science, 5,210 from Scopus, and 1,615 from PubMed. After applying predefined exclusion criteria, we removed irrelevant studies, non-academic items (e.g., conference abstracts, books, and editorials), and duplicate records. Using the bibliometrix package to merge and deduplicate the three datasets, a total of 4,954 eligible publications were retained for subsequent bibliometric analyses. Details of the study selection and data synthesis process are shown in Supplementary Figure S1 and Supplementary Table S2.

To further verify the robustness of the search strategy, we conducted a supplementary sensitivity search. Based on the main search strategy, we added frailty-related alternative keywords with broader conceptual boundaries, including “sarcopenia,” “muscle weakness,” “functional decline,” “physical function decline,” and “disability.” The results showed that the expanded search strategy substantially increased the number of retrieved records: from 1,853 to 8,901 in the Web of Science Core Collection (WoSCC), from 5,210 to 23,913 in Scopus, and from 1,615 to 7,720 in PubMed. Overall, the total number of retrieved records increased from 8,678 to 40,534. These findings suggest that although broadly incorporating these alternative keywords improved search sensitivity, it may also introduce a large number of records related only to sarcopenia, general functional decline, disability, or physical dysfunction, rather than frailty research in the strict sense. Therefore, the final analysis was still based on the records retrieved using the main search strategy centered on frail^*^/frailty. The results of the sensitivity search are presented in Supplementary Table S1.

### 3.2 Annual publication trend of hypertension–frailty research

**Figure 1:**
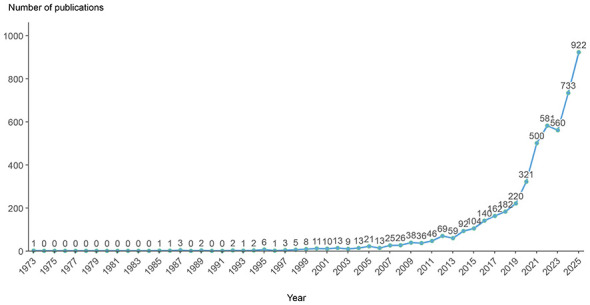
Annual publication trends in hypertension and frailty research.

Figure 1 presents the annual publication output on hypertension and frailty from 1973 to 2025. The number of publications was minimal from 1973 to 2000, remaining close to zero. After 2000, the publication output increased gradually and then showed rapid growth after 2010, with a particularly steep rise after 2015. By 2023, the annual number of publications approached 1,000, indicating sustained expansion and increasing attention to this research area.

### 3.3 Country-level distribution and collaboration patterns

As shown in Table 1, the global research output on hypertension and frailty was led by the United States (*n* = 1,086, 21.9%), followed by China (*n* = 542, 10.9%) and Italy (*n* = 379, 7.7%). Japan (*n* = 341, 6.9%) and the United Kingdom (*n* = 297, 6.0%) also ranked among the top five most productive countries. Together, these countries accounted for a substantial proportion of the total publications in this field. In terms of collaboration type, most publications were single-country publications (SCPs) across all major contributing countries, indicating that domestic research remained the dominant pattern. The United States had the highest number of SCPs (1010) and Multiple Country Publications (MCPs) (76), suggesting both strong independent productivity and active international collaboration. China also showed high productivity with 511 SCPs and 31 MCPs, but a relatively low MCPs proportion (5.7%), indicating that its output was mainly driven by domestic collaborations. Among the high-output countries, several European and Oceanian countries exhibited relatively stronger international collaboration. The Netherlands (MCP% = 18.2%), Australia (16.0%), United Kingdom (14.8%), France (14.6%), and Canada (14.5%) showed comparatively high proportions of multi-country publications. Notably, although Switzerland had a lower publication volume (*n* = 45), it had the highest MCPs proportion (20.0%), reflecting a high degree of international cooperation. In contrast, countries such as Japan (3.8%), Korea (3.3%), Turkey (2.4%), and India (2.0%) had relatively low MCPs proportions, suggesting more limited cross-national collaboration in this research area. Overall, these findings indicate that research on hypertension and frailty is concentrated in a few high-income countries, with substantial heterogeneity in international collaboration intensity across countries.

**Table 1:** Distribution and collaboration patterns of hypertension and frailty-related literature by country.

Country	Articles	Articles %	SCP	MCP	MCP %
USA	1,086	21.9	1,010	76	7
China	542	10.9	511	31	5.7
Italy	379	7.7	329	50	13.2
Japan	341	6.9	328	13	3.8
United Kingdom	297	6	253	44	14.8
Canada	193	3.9	165	28	14.5
Spain	159	3.2	151	8	5
Australia	150	3	126	24	16
France	130	2.6	111	19	14.6
Korea	120	2.4	116	4	3.3
Germany	98	2	88	10	10.2
Brazil	96	1.9	92	4	4.2
Turkey	85	1.7	83	2	2.4
Netherlands	77	1.6	63	14	18.2
Poland	64	1.3	61	3	4.7
Singapore	53	1.1	45	8	15.1
India	49	1	48	1	2
Switzerland	45	0.9	36	9	20
Sweden	41	0.8	36	5	12.2
Israel	37	0.7	35	2	5.4

SCP, single-country studies; MCP, multi-country collaboration.

### 3.4 Top productive authors, journals, and institutions

**Figure 2:**
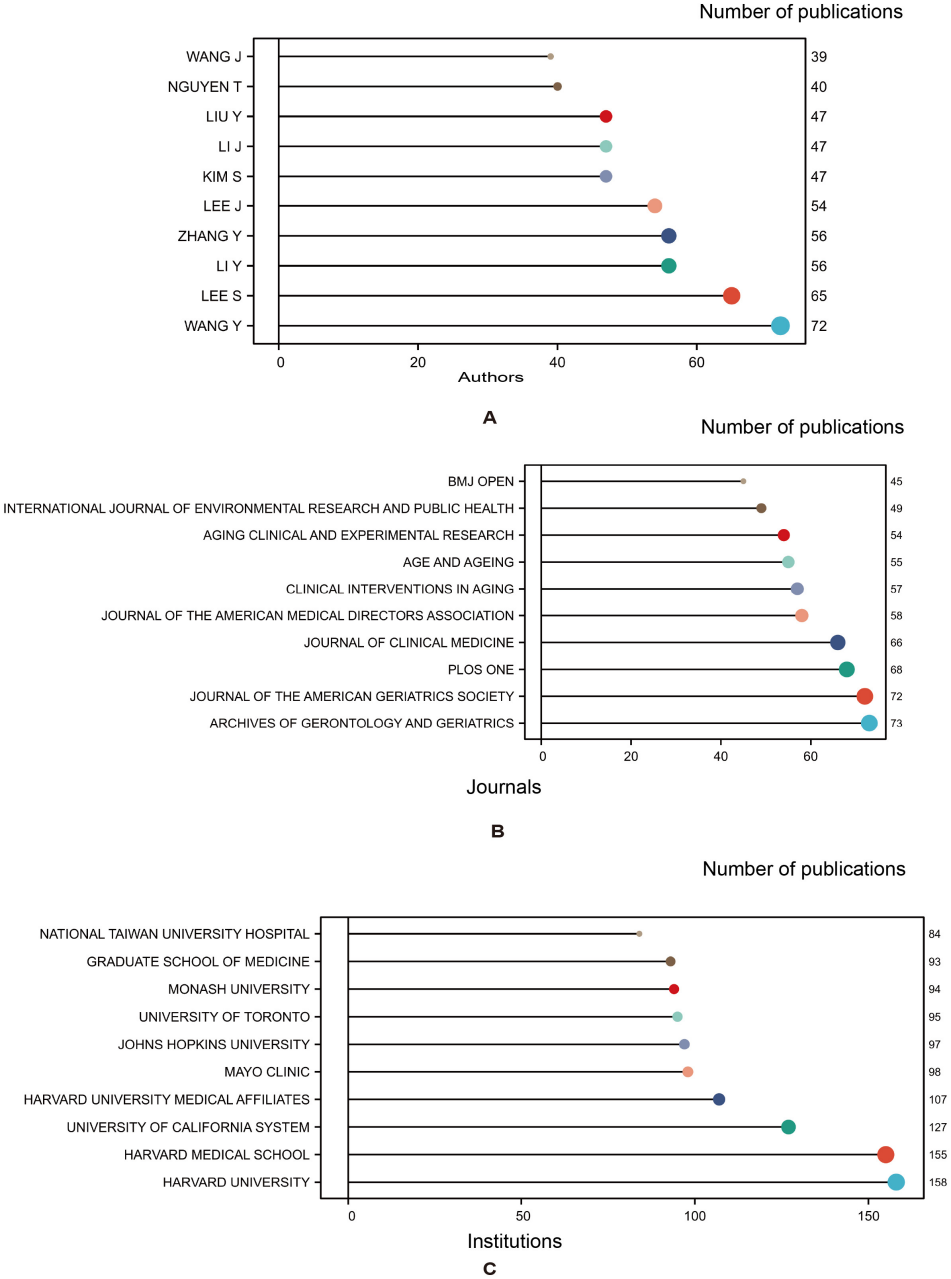
Contributions of authors, journals, and institutions in hypertension and frailty research. **(A)** (Authors): identifies leading researchers in the field and shows the diversity of contributors across different regions. **(B)** (Journals): highlights which journals are most frequently publishing research on hypertension and frailty, which is useful for understanding where the bulk of this research is disseminated. **(C)** (Institutions): displays the leading institutions contributing to research in this field, reflecting the importance of certain academic and medical centers in advancing knowledge on hypertension and frailty.

Figure 2 displays the top 10 authors, journals, and institutions in hypertension–frailty research. In Figure 2A, Wang Y was the most prolific author with 72 publications, followed by Lee SJ (65) and Li Y (56). Figure 2B shows that Archives of Gerontology and Geriatrics published the largest number of related papers (73), followed by the Journal of the American Geriatrics Society and PLOS ONE. Figure 2C indicates that Harvard University was the leading institution (158 publications), followed by Harvard Medical School. Overall, these findings highlight the major contributors and highly productive institutions shaping the field.

### 3.5 Keyword analysis based on bibliometrix

**Figure 3:**
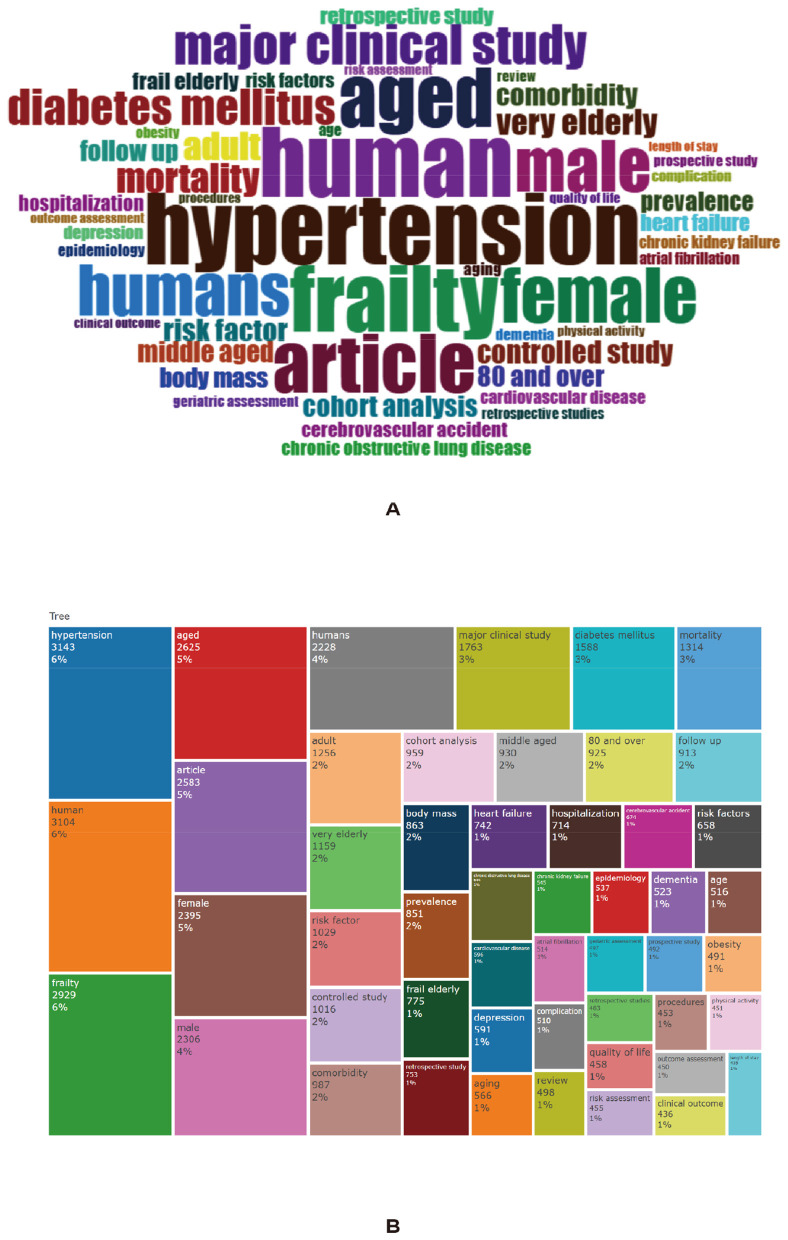
Keyword analysis of hypertension and frailty research. **(A)** Presents a word cloud showing the most frequent keywords related to hypertension and frailty research. **(B)** Displays a treemap representation of the same keywords, showing their respective frequency as percentages of the total.

Figure 3 summarizes the major keywords and their distributions in the hypertension–frailty literature. The word cloud (Figure 3A) highlights “Hypertension” and “Frailty” as central topics. High-frequency terms such as “Human” and “Aged” indicate that study populations predominantly involve older adults. Additional frequently occurring keywords include “Major clinical study,” “Risk factor,” and “Diabetes mellitus,” reflecting the multifactorial context of hypertension and frailty research. The treemap (Figure 3B) further quantifies keyword distributions, showing that “Hypertension” and “Frailty” each accounted for 6% of all records, compared with “Diabetes mellitus” (3%) and “Prevalence” (2%), indicating strong thematic concentration around the core concepts.

### 3.6 Institutional collaboration and keyword co-occurrence networks based on VOSviewer

**Figure 4:**
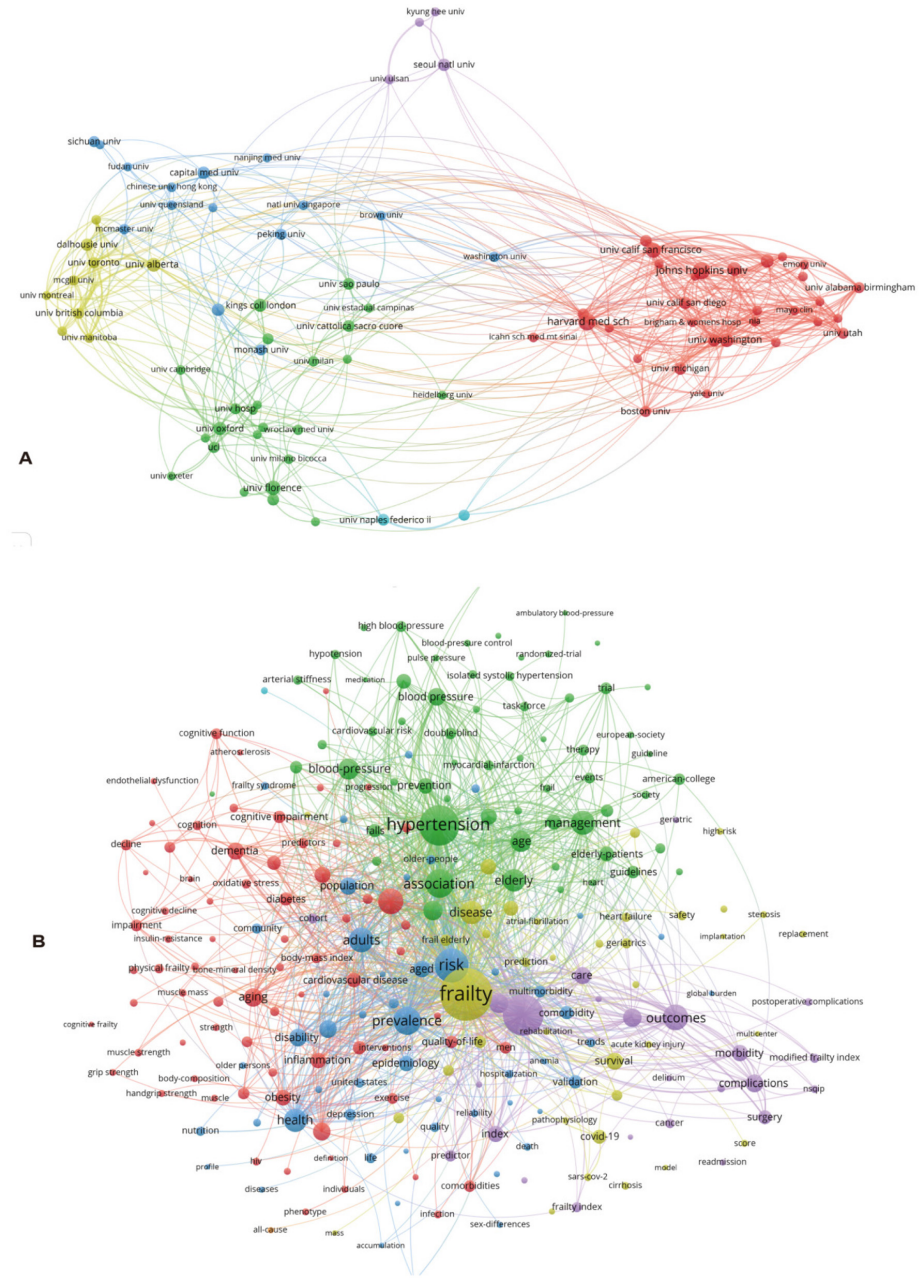
Institutional collaboration network and keyword co-occurrence map in hypertension–frailty research (VOSviewer visualization). **(A)** Institutional collaboration network. Nodes represent institutions, with larger nodes indicating higher publication output. Links denote collaborative relationships; thicker/denser links indicate stronger collaboration. Colors indicate collaboration clusters (communities). **(B)** Keyword co-occurrence network. Nodes represent keywords, with larger nodes indicating higher occurrence frequency. Links denote co-occurrence relationships; thicker links indicate stronger co-occurrence. Colors indicate thematic clusters, highlighting major research foci such as hypertension management and blood pressure monitoring, frailty assessment and functional metrics (e.g., grip strength, gait speed, body composition), and outcomes/complications (e.g., hospitalization, mortality, acute kidney injury).

Figure 4A (institutional collaboration network) illustrates the collaboration structure among high-producing institutions. Nodes represent institutions (larger nodes indicate higher productivity), links represent collaborations (denser/thicker links indicate stronger collaboration), and colors indicate collaboration clusters. Six clusters were identified. Cluster 1 (31 institutions) was dominated by U.S. institutions with dense internal collaborations (e.g., Harvard Med Sch/Harvard Univ, Johns Hopkins Univ, UCSF, UAB, Stanford, Yale). Cluster 2 (24 institutions) primarily comprised European institutions (e.g., Karolinska Inst, King's College London, Heidelberg Univ, Leiden Univ, Lund Univ). Cluster 3 (15 institutions) mainly included institutions from China and the Asia–Pacific region and connected with some North American institutions (e.g., Peking Univ, Fudan Univ, Nanjing Med Univ, Chinese Acad Med Sci, CUHK, NUS, Univ Sydney/Queensland/UNSW, Monash). Cluster 4 (9 institutions) consisted predominantly of Canadian institutions (e.g., Univ Toronto, McGill, UBC, Univ Alberta). Cluster 5 (4 institutions) was largely composed of Korean institutions (e.g., Seoul Natl Univ, Kyung Hee Univ, Sungkyunkwan Univ, Univ Ulsan). Cluster 6 (2 institutions) included Albert Einstein Coll Med and Univ Naples Federico II. Overall, extensive cross-cluster links suggest a notable level of international collaboration.

Figure 4B (keyword co-occurrence network) depicts the knowledge structure and research hotspots. “Frailty” and “Hypertension” were located at the center of the network, indicating their core roles. Surrounding high-frequency and strongly connected keywords included “risk,” “prevalence,” “association,” “management,” and “outcomes,” suggesting that the literature mainly focuses on epidemiological associations, risk evaluation, clinical management, and prognostic outcomes. The clustering results further indicated multiple thematic domains: one cluster emphasized blood pressure monitoring, antihypertensive treatment, and guidelines (e.g., blood pressure, ambulatory blood pressure, antihypertensive drug, guideline); another cluster focused on frailty-related outcomes, complications, and hospitalization/mortality risks (e.g., outcomes, complications, hospitalization, death, acute kidney injury, delirium, heart failure); and a third cluster highlighted frailty phenotypes, body composition, functional indicators, and biological mechanisms (e.g., body composition, grip strength, gait speed, biomarkers, inflammation). In addition, clusters related to cognitive function/dementia and perioperative/postoperative complications were observed, suggesting extension of the field into neurocognitive and surgical risk-management contexts.

Based on the collaboration network analysis, we further standardized institutional publication contributions to more clearly reflect the relative output levels of major institutions. To reduce duplicate counting caused by differences in institutional name spelling, abbreviations, and affiliation hierarchies across databases, institutional names were harmonized in this study. Based on the 3,724 publications with available institutional information, Harvard University ranked first by publication output, with 158 publications, accounting for 4.24% of the final dataset. It was followed by Harvard Medical School (155 publications, 4.16%), the University of California System (127 publications, 3.41%), Harvard University Medical Affiliates (107 publications, 2.87%), and Mayo Clinic (98 publications, 2.63%). Johns Hopkins University, the University of Toronto, Monash University, Graduate School of Medicine, and National Taiwan University Hospital were also among the top 10 institutions.

Overall, the top 10 institutions contributed 1,108 publications, accounting for 29.75% of the final included publications, indicating that institutional output in this field is mainly concentrated in high-level universities and medical research institutions in North America, Asia, and Australia. It should be noted that institutional contributions were counted using the full counting method; therefore, a multi-institutional collaborative paper could be counted for multiple institutions. The corresponding percentages were used to reflect the relative output intensity at the institutional level rather than mutually exclusive proportions. Detailed results are presented in Supplementary Table S5.

### 3.7 Research hotspots revealed by CiteSpace keyword clustering and citation burst analysis

**Figure 5:**
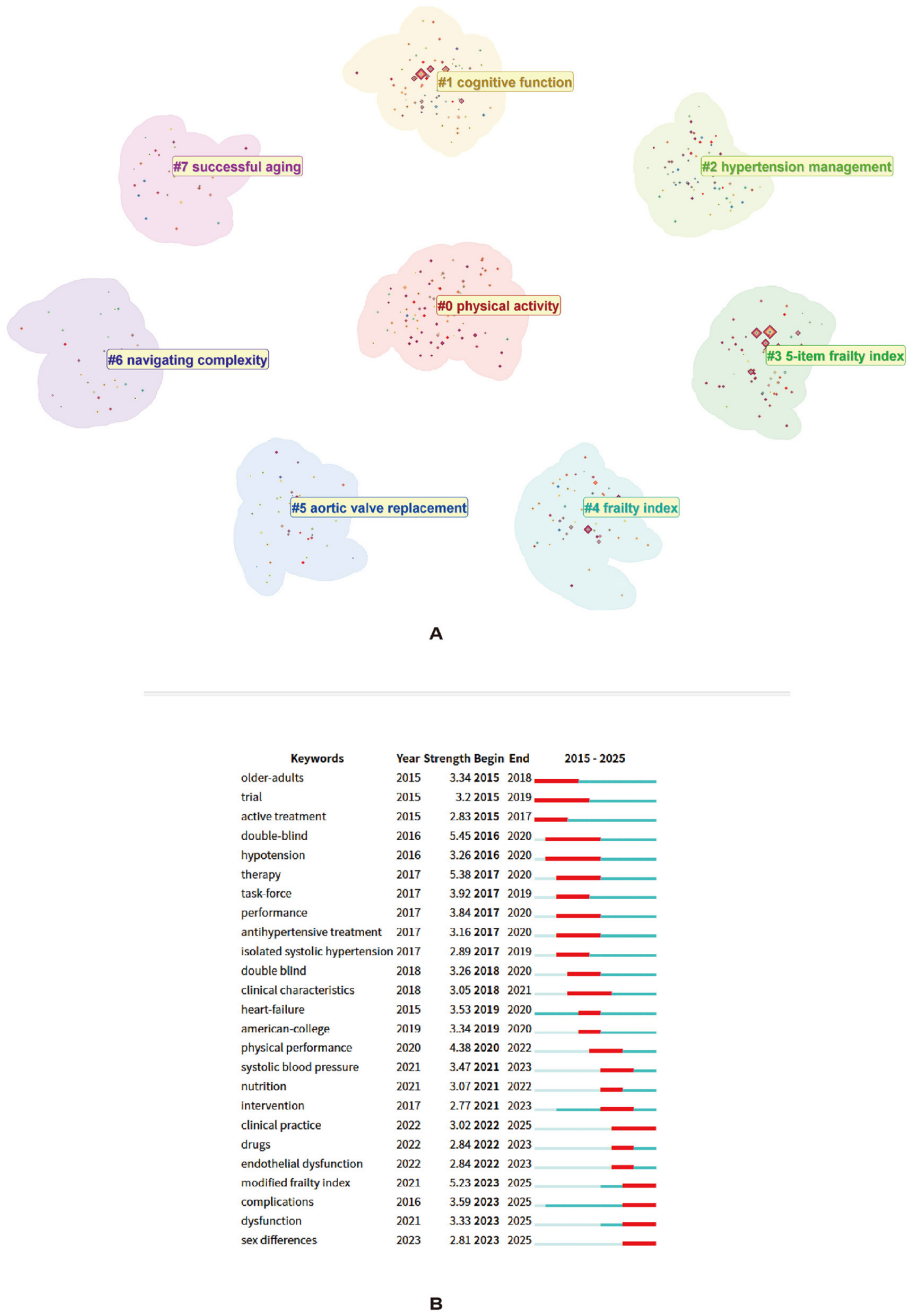
Keyword clustering and citation-burst analysis of hypertension–frailty research (CiteSpace visualization, 2015–2025). **(A)** Keyword clustering map. Each colored region represents a keyword cluster (topic community). Major clusters include #0 Physical activity, #1 Cognitive function, #2 Hypertension management, #3 5-item frailty index, #4 Frailty index, #5 Aortic valve replacement, #6 Navigating complexity, and #7 Successful aging, reflecting the main thematic structure of the field. **(B)** Top 25 keywords with the strongest citation bursts. The table lists burst strength and the start/end years of each burst. Red segments indicate the time window during which a keyword experienced a rapid increase in citations, highlighting emerging or intensifying research frontiers during 2015–2025.

As shown in Figure 5A, the keyword co-occurrence network was grouped into eight major clusters, reflecting the main thematic structure of research on hypertension and frailty. The largest cluster was #0 “physical activity”, indicating that exercise-related interventions and functional maintenance are central topics in this field. Other prominent clusters included #1 “cognitive function”, #2 “hypertension management”, #3 “5-item frailty index”, and #4 “frailty index”, suggesting sustained attention to cognitive outcomes, blood pressure management strategies, and frailty assessment tools. In addition, #5 “aortic valve replacement” points to a clinically oriented subfield focusing on cardiovascular procedures and perioperative frailty evaluation, while #6 “navigating complexity”, and #7 “successful aging” reflect broader geriatric care and healthy aging perspectives.

Figure 5B presents the top 25 keywords with the strongest citation bursts from 2015 to 2025, revealing the evolution of research hotspots over time. Early burst keywords (mainly 2015–2020) included “older-adults,” “trial,” “active treatment,” “double-blind,” “hypotension,” “therapy,” “antihypertensive treatment,” and “isolated systolic hypertension,” indicating an early emphasis on clinical trials, treatment strategies, and blood pressure control. During the middle period, terms such as “physical performance,” “systolic blood pressure,” “nutrition,” and “intervention” emerged, reflecting increasing interest in functional outcomes and multidimensional management. Notably, the most recent and ongoing burst keywords (continuing to 2025) include “clinical practice,” “modified frailty index,” “complications,” “dysfunction,” and “sex differences,” suggesting that current research is shifting toward real-world clinical application, risk stratification using frailty instruments, complication prediction, and subgroup heterogeneity. Among these, “modified frailty index” showed a relatively strong and recent burst, highlighting its growing importance in contemporary research. The CiteSpace time-slice sensitivity analysis showed that the main burst themes remained generally consistent under the 1-year, 2-year, and 3-year time-slice settings. These themes included hypertension management, frailty assessment, older adults, clinical trials, physical function, cardiovascular outcomes, and nutrition-related topics, suggesting that the citation burst detection results had good stability. The relevant results are presented in Supplementary Table S3.

The VOSviewer threshold sensitivity analysis showed that setting the minimum keyword occurrence threshold to 10 achieved a good balance among thematic coverage, network complexity, and map interpretability. A threshold of 5 included more keywords but resulted in a denser network, whereas a threshold of 15 produced a more simplified network but may have reduced some medium-frequency themes. Therefore, this study adopted a threshold of 10 as the main analytical parameter. The relevant results are presented in Supplementary Table S4.

### 3.8 Citation leadership in hypertension–frailty research: top authors, journals, and countries

**Figure 6:**
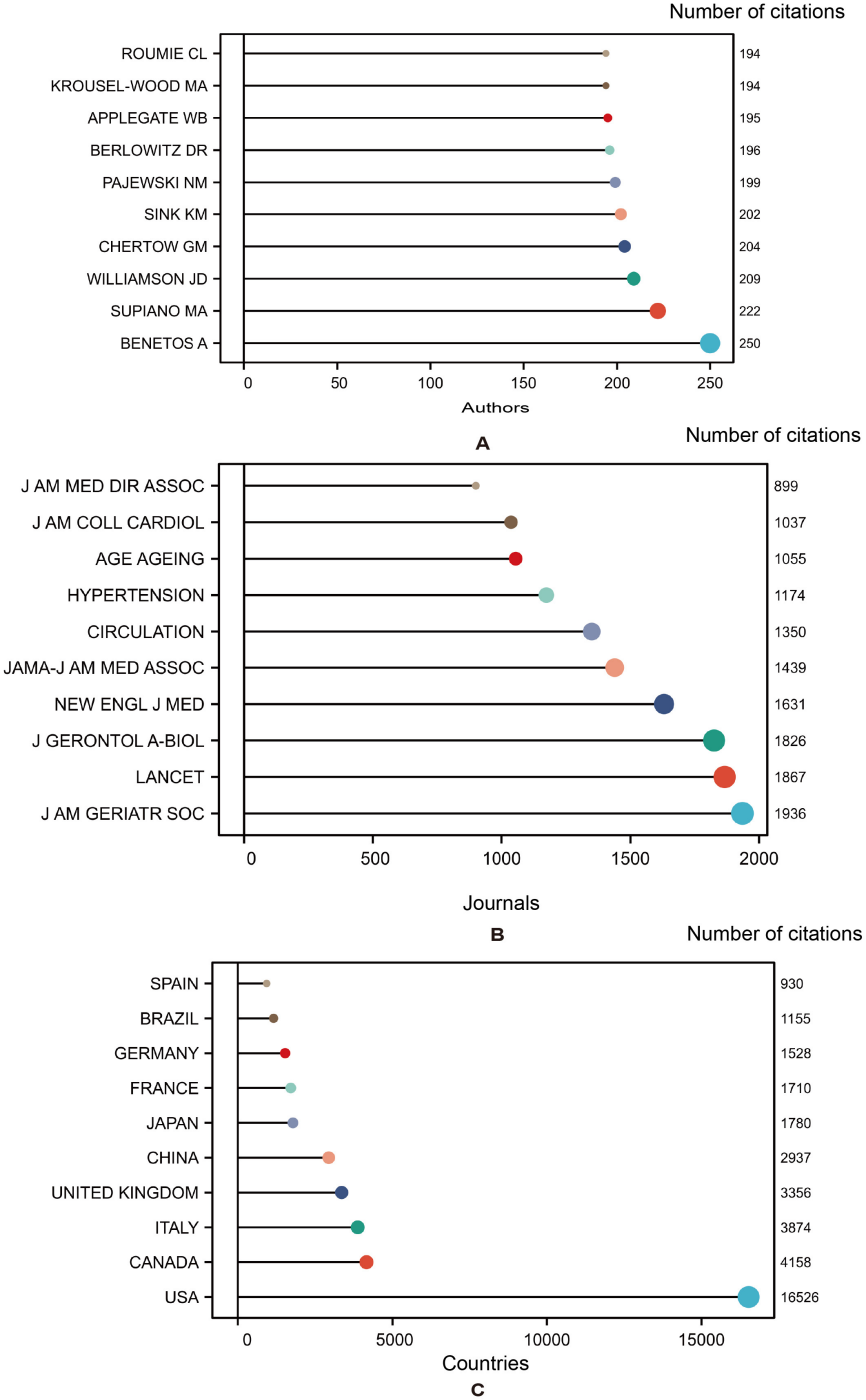
Top 10 most cited authors, journals, and countries in hypertension and frailty research. **(A)** Shows the top 10 most cited authors in the field of hypertension and frailty research. **(B)** Presents the top 10 most cited journals in the same research field. **(C)** Illustrates the top 10 most cited countries.

Figure 6 summarizes the most-cited contributors in hypertension–frailty research at the levels of authors (A), journals (B), and countries (C). Figure 6A (Authors). Citations are relatively concentrated among a small group of senior investigators. Benetos A ranks first (250 citations), followed by Supiano MA (222) and Williamson JD (209). The remaining top authors cluster in a narrower range—from Chertow GM (204) and Sink KM (202) to Pajewski NM (199), Berlowitz DR (196), Applegate WB (195), and Krousel-Wood MA/Roumie CL (both 194), suggesting broadly comparable citation influence among the top 10. Figure 6B (Journals). Citations are dominated by major geriatrics and high-impact general/cardiovascular journals. The Journal of the American Geriatrics Society leads (1,936 citations), followed by The Lancet (1,867) and Journal of Gerontology: Series A (1,826). Other highly cited outlets include New England Journal of Medicine (1,631), JAMA (1,439), Circulation (1,350), and Hypertension (1,174), indicating that influential evidence in this field is published across both geriatrics and cardiovascular medicine. Figure 6C (Countries). Citation impact is strongly skewed toward the USA, which ranks first by a wide margin (16,526 citations). A second tier includes Canada (4,158), Italy (3,874), and the United Kingdom (3,356), followed by China (2,937). Other countries (Japan, France, Germany, Brazil, Spain) contribute smaller but still notable citation totals, reflecting a broadly international knowledge base with clear leadership from North America and Western Europe.

### 3.9 Global and local citation analyses

Table 2 lists the top 10 most globally cited papers in the hypertension–frailty field. The most cited publication was Salim Yusuf's PURE prospective cohort study, “Modifiable risk factors, cardiovascular disease, and mortality in 155,722 individuals from 21 high-income, middle-income, and low-income countries (PURE): a prospective cohort study” (Lancet, 2020), with 1,156 global citations (22). This was followed by Giovanni Guaraldi's paper, “Premature age-related comorbidities among HIV-infected persons compared with the general population” (Clin Infect Dis, 2011), with 1,048 citations (23), and Jeff D Williamson's SPRINT trial paper, “Intensive vs. Standard Blood Pressure Control and Cardiovascular Disease Outcomes in Adults Aged ≥75 Years: A Randomized Clinical Trial” (JAMA, 2016), with 988 citations (24). Other highly cited publications included L. P. Fried's Cardiovascular Health Study paper (JAMA, 1998; 895 citations) (25), and Rodés-Cabau's transcatheter aortic valve implantation study (J Am Coll Cardiol, 2010; 840 citations) (26).

**Table 2:** Top 10 most cited articles on hypertension and frailty: global citation overview.

Number	First author	Article name	Journal name	Year	Global citations
1	Salim Yusuf	Modifiable risk factors, cardiovascular disease, and mortality in 155 722 individuals from 21 high-income, middle-income, and low-income countries (PURE): a prospective cohort study	Lancet	2020	1,156
2	Giovanni Guaraldi	Premature age-related comorbidities among HIV-infected persons compared with the general population	Clin Infect Dis	2011	1,048
3	Jeff D. Williamson	Intensive vs. standard blood pressure control and cardiovascular disease outcomes in adults aged ≥75 years: a randomized clinical trial	JAMA	2016	988
4	L. P. Fried	Risk factors for 5-year mortality in older adults: the cardiovascular health study	JAMA	1998	895
5	Josep Rodés-Cabau	Transcatheter aortic valve implantation for the treatment of severe symptomatic aortic stenosis in patients at very high or prohibitive surgical risk: acute and late outcomes of the multicenter Canadian experience	J Am Coll Cardiol	2010	840
6	Ulrich Klotz	Pharmacokinetics and drug metabolism in the elderly	Drug Metab Rev	2009	587
7	Francesco Landi	Sarcopenia and mortality risk in frail older persons aged 80 years and older: results from ilSIRENTE study	Age Aging actions Search in PubMed Search in NLM Catalog Add to Search.	2013	482
8	J. N. Morris	Walking to health	Sports Med	1997	464
9	Wei Xu	Meta-analysis of modifiable risk factors for Alzheimer's disease	J Neurol Neurosurg Psychiatry	2015	460
10	Laura Perna	Epigenetic age acceleration predicts cancer, cardiovascular, and all-cause mortality in a German case cohort	Clin Epigenetics	2016	420

Supplementary Table S6 presents the top 10 locally cited papers within the hypertension–frailty literature. The most locally cited publication was Williamson's SPRINT trial paper (JAMA, 2016), with 142 local citations (24). It was followed by Jane Warwick's paper “No evidence that frailty modifies the positive impact of antihypertensive treatment in very elderly people” (BMC Med, 2015), with 92 local citations (27), and Michelle C. Odden's paper “Rethinking the association of high blood pressure with mortality in elderly adults: the impact of frailty” (Arch Intern Med, 2012), with 88 local citations (28). Other locally influential works included Benetos's PARTAGE study (JAMA Intern Med, 2015; 73 local citations) (29) and “Hypertension Management in Older and Frail Older Patients” (Circ Res, 2019; 69 local citations) (30) Collectively, these studies have exerted substantial influence on both international and local citation landscapes in this field.

## 4 Discussion

This bibliometric study, based on a systematic retrieval of publications from WoSCC, Scopus, and PubMed, delineated the overall development trajectory of research on hypertension and frailty, and mapped the major contributors (countries/regions, institutions, journals, and authors), the thematic knowledge structure reflected by keywords, as well as the global and local citation landscapes.

### 4.1 Growth of the field and potential drivers

Across 1973–2025, the volume of publications related to “hypertension” and “frailty” increased substantially. The output remained minimal from 1973 to 2000, began to rise steadily after 2000, accelerated after 2010, and showed a particularly sharp increase after 2015. By 2023, annual publications approached 1,000, indicating that this interdisciplinary topic has gained sustained and growing attention. This expansion is likely driven by multiple converging factors. First, global population aging has increased the prevalence of both hypertension and frailty among older adults, thereby intensifying clinical and public-health needs and stimulating research production (31, 32). Second, the clinical interplay between hypertension and multidimensional frailty has become increasingly recognized; their bidirectional relationship may involve complex biological pathways, which has further attracted academic interest (33). Third, the rising burden of multimorbidity and chronic diseases, along with increasing interdisciplinary collaboration, may also have facilitated the rapid development of this field (34). Collectively, these factors have shaped the strong growth observed in research output and have provided a robust foundation for future investigations.

### 4.2 Geographic productivity and international collaboration

In terms of national and regional contributions, the United States was the leading producer, accounting for over 20% of publications, followed by China and Italy. The United States showed a relatively low proportion of MCPs, whereas Italy and other European countries exhibited higher MCPs proportions, suggesting relatively stronger cross-border collaboration in Europe. In particular, Switzerland presented the highest MCPs proportion (20%), indicating an active trend toward international collaboration in this domain, especially in European research networks. Nevertheless, the country ranking and collaboration metrics should be interpreted with caution. First, reliance on major English-language databases may underestimate contributions from non-English-speaking countries (e.g., Germany and Russia). Second, collaboration frequency is derived from co-authorship links between countries, and multinational collaborations may be simplified into pairwise relationships, potentially underestimating the overall contribution of multilateral collaborations. Third, national and institutional influence may vary over time; cumulative rankings may mask temporal dynamics. Future bibliometric work may mitigate these issues by incorporating multi-language sources and conducting time-series analyses (35).

### 4.3 Key contributors: authors, journals, and institutions

Regarding author productivity, Wang Y was the most prolific researcher in this field (72 publications). In journal distribution, Archives of Gerontology and Geriatrics published the highest number of related studies, highlighting its influence in disseminating evidence on aging, frailty, and cardiovascular risk. At the institutional level, Harvard University and Harvard Medical School led the field in publication output (158 publications and similarly high productivity), underscoring the pivotal role of top academic centers in advancing research at the interface of geriatrics and cardiovascular medicine.

### 4.4 Thematic structure and emerging hotspots revealed by keywords

Keyword analyses identified major research hotspots and the underlying knowledge structure of hypertension–frailty research. Co-occurrence and clustering results indicated that the field primarily focuses on hypertension management, frailty assessment tools, health management in older populations, and clinical outcomes. “Hypertension” and “Frailty” were the most central keywords, reflecting their foundational importance for older adults' health. With increasing aging-related health challenges, research attention has extended beyond hypertension alone, as other chronic conditions, marital status, lifestyle factors, physical activity, and obesity have also been increasingly recognized as correlates of frailty (36, 37). A key feature of this literature is the growing prominence of frailty assessment instruments, particularly the Frailty Index (FI). FI quantifies accumulated health deficits (including diseases, disabilities, signs, and symptoms). Based on the standard procedure proposed by Searle and further developed by Hakeem and colleagues, FI typically includes 49 deficits spanning cognition, dependency, depressive symptoms, comorbidities, healthcare utilization, access to care, physical function, anthropometrics, and laboratory parameters (38). Evidence suggests that FI is an independent predictor and can capture health status information across multiple physiological systems (5, 39). With increasing adoption of FI (including the 5-item frailty index) in both research and practice, frailty metrics have become more integrated into hypertension research and geriatric health management. This methodological evolution has likely contributed to the rapid growth of publications in this domain, reflecting the central role of frailty assessment in older-adult care and in understanding hypertension–frailty comorbidity.

In addition, the high frequency of keywords such as “risk” and “prevalence” indicates that researchers have emphasized not only clinical management but also epidemiological associations and risk stratification. Prior evidence suggests that frailty prevalence in older adults ranges from 7% to 28%, is higher in women, and increases with age (40, 41). This aligns with the broader context of multimorbidity in older populations, in which the interaction between hypertension and frailty can critically shape health outcomes. Notably, keywords related to functional indicators—including “body composition,” “grip strength,” and “gait speed”—appeared prominently. These markers reflect different dimensions of physiological reserve and functional decline: body composition captures structural substrates such as muscle and fat distribution; grip strength represents global muscle strength and neuromuscular function; and gait speed integrates motor control, cardiopulmonary endurance, and balance. Existing studies have reported significant correlations between daily functional capacity and anthropometric measures (e.g., body mass index and arm circumference) as well as grip strength in community-dwelling older adults; moreover, lower grip strength is closely associated with sarcopenia and has been linked to increased fall risk and frailty (42). Together, these findings support a pathway in which changes in body composition (e.g., muscle mass loss) may contribute to functional decline via reduced strength and mobility, ultimately affecting independence in daily activities. Therefore, body composition, grip strength, and gait speed serve not only as important indicators of frailty risk (potential functional biomarkers) but also as core elements of comprehensive geriatric assessment. In older adults with hypertension, incorporating these measures into routine follow-up may better characterize physical reserve and functional status, enabling earlier identification of risks such as disability, falls, and hospitalization and informing individualized interventions and long-term management.

The appearance of keywords such as “physical activity” and “successful aging” further suggests that research attention is shifting from single-parameter blood pressure control toward function-preserving strategies and healthy aging promotion. At the evidence level, a systematic review by Elisa María Garrido- Ardila et al. reported that exercise interventions can sustainably improve physical function and muscle strength in hemodialysis patients (43), implying that even in vulnerable populations with multimorbidity and low reserve, exercise may yield stable functional benefits. Moreover, randomized evidence also supports actionable intervention pathways: Neng Pan et al. found that a multicomponent Otago Exercise Program (OEP) combined with resistance training (RT) improved sarcopenia-related indicators among prefrail nursing-home residents, suggesting a practical “strengthening—function improvement” intervention chain. Importantly, frailty status may not reverse synchronously in the short term; the phenomenon of “functional improvement preceding frailty reversal” may be attributable to limited intervention duration, insufficient intensity, or relatively narrow intervention components (e.g., lack of integrated nutrition support, psychological/cognitive training, and multimorbidity/medication management). Nevertheless, improvements in muscle mass and function remain clinically meaningful, as they may reduce falls and disability risks and enhance endurance and daily activity capacity, thereby supporting quality-of-life improvement and potentially facilitating longer-term frailty mitigation (44). In line with these observations, citation burst analysis showed that keywords such as “frailty index” and “hypertension management” have surged rapidly in recent years, indicating that the field is moving from descriptive association studies toward a more advanced stage characterized by quantifiable assessment tools and management/intervention-oriented research. This evolution highlights the growing emphasis on integrating frailty assessment into hypertension care pathways and optimizing interventions based on functional outcomes.

### 4.5 From risk stratification to integrated intervention: clinical implications of research on hypertension and frailty

In recent years, a series of studies has further enriched the clinical translational evidence at the intersection of hypertension and frailty. First, increasing attention has been paid to the combined predictive value of metabolic abnormalities and frailty assessment. The triglyceride-glucose index (TyG) is an effective surrogate marker of insulin resistance, whereas the FI reflects the cumulative decline in physiological function. A recent study further proposed the TyG-frailty index (TyGFI), an integrated indicator, and examined its association with the risk of cardiovascular disease and stroke based on two representative cohorts: the China Health and Retirement Longitudinal Study (CHARLS) and the National Health and Nutrition Examination Survey (NHANES). The results showed that higher TyGFI levels were associated with older age, an unfavorable metabolic profile, and a higher prevalence of hypertension, diabetes, and dyslipidemia. After full adjustment for demographic characteristics, clinical factors, and lifestyle factors, participants in the highest TyGFI quartile still had significantly increased risks of cardiovascular disease and stroke, with a consistent dose–response relationship. This study suggests that TyGFI, by integrating metabolic burden and functional decline, may provide a more comprehensive assessment of cardiovascular risk in older adults than either metabolic indicators or frailty assessment alone. Therefore, in older adults with hypertension, risk stratification should not rely solely on blood pressure levels but should also consider insulin resistance, metabolic abnormalities, frailty severity, and functional reserve, so as to identify individuals at high risk of cardiovascular disease and stroke earlier and provide evidence for individualized prevention and integrated management (45). Second, cognitive impairment is an important clinical issue in patients with both hypertension and frailty. A study by Mone et al. showed that choline bitartrate combined with vitamin B12 improved cognitive function in older hypertensive patients with cognitive frailty, providing preliminary evidence for the application of nutritional intervention in this population (46). Regarding functional interventions, exercise training, particularly high-intensity interval training (HIIT), may provide an effective non-pharmacological management strategy for older patients with hypertension. However, its antihypertensive effect may be influenced by individual factors such as vascular function, autonomic regulation, and exercise capacity. A randomized study involving 80 older hypertensive patients showed that 12 weeks of HIIT significantly reduced systolic and diastolic blood pressure and improved several biological indicators related to blood pressure regulation. Specifically, after HIIT, Salusin-α levels increased, whereas Salusin-β, the Salusin-β/α ratio, and carotid-femoral pulse wave velocity (cfPWV) decreased, accompanied by improvements in heart rate variability (HRV) indicators. Further analysis showed that Salusin-β, low-frequency power (LF), high-frequency power (HF), and peak oxygen uptake (VO_2_ peak) explained most of the variation in the systolic blood pressure response, whereas Salusin-β, cfPWV, VO_2_ peak, and HF explained most of the variation in the diastolic blood pressure response. These findings suggest that the blood pressure-lowering benefits of HIIT are not uniform but may be jointly influenced by vascular stiffness, autonomic function, exercise tolerance, and Salusin-related pathways. Therefore, in older adults with hypertension complicated by frailty or reduced functional reserve, future exercise prescriptions should not be designed solely according to age or blood pressure level. Instead, they should comprehensively consider frailty severity, physical functional reserve, baseline blood pressure, vascular function, and exercise tolerance, thereby enabling more individualized and safer functional interventions (47, 48). In addition, frailty status is not a static or single-dimensional clinical characteristic, and its role in blood pressure target setting and intensive antihypertensive treatment has received increasing attention. Current hypertension guidelines generally emphasize individualized blood pressure management according to age, comorbidities, and treatment tolerance. However, chronological age alone may not accurately reflect patients' biological heterogeneity. A recent study based on participants from the Systolic Blood Pressure Intervention Trial (SPRINT) and the Action to Control Cardiovascular Risk in Diabetes (ACCORD) further suggested that FI, as a multidimensional indicator of biological aging based on the cumulative deficit model, may better distinguish the optimal systolic blood pressure control range for different patients than chronological age. This study calculated *FI* using a 31-item Rockwood cumulative deficit model and defined frailty as *FI* > 0.21. It also compared the association between time in target range (TTR) for different systolic blood pressure ranges and major adverse cardiovascular events. The results showed a J-shaped relationship between mean systolic blood pressure and major adverse cardiovascular events, and this risk stratification was more evident when patients were classified by frailty status than by age. Among frail individuals, a longer time with systolic blood pressure maintained within the range of 110–140 mmHg was associated with a lower risk of major adverse cardiovascular events, whereas among non-frail individuals, a longer time with systolic blood pressure below 130 mmHg was associated with a greater risk reduction. This study indicates that frailty severity may influence the benefit–risk balance of intensive antihypertensive treatment. Therefore, when determining blood pressure management targets for older hypertensive patients, clinicians should dynamically assess frailty status, renal function, fall risk, hypotension risk, and treatment tolerance, rather than making decisions based solely on chronological age or a single blood pressure value (49). Metabolic factors also warrant attention. A study by Monteforte showed that hyperglycemia may promote the transition from prefrailty to frailty in older adults (50). Further research indicated that prediabetes may increase the risk of developing frailty among prefrail older adults with hypertension, whereas metformin may have a certain protective effect (51). In patients with chronic CKD, Santulli et al. also found that frail older hypertensive patients with prediabetes and CKD had risks of organ damage and cognitive decline, suggesting that kidney injury, metabolic abnormalities, and cognitive impairment may jointly constitute a high-risk clinical phenotype (52, 53). Regarding nutritional supplementation, L-arginine combined with vitamin C has been reported to improve physical frailty in older adults with hypertension, suggesting that endothelial function, oxidative stress, and muscle function may be potential intervention targets (54). Overall, these studies are consistent with the individualized management concept emphasized in current guidelines for older and very old patients with hypertension: blood pressure targets should not be determined solely by age but should comprehensively consider frailty severity, functional status, comorbidities, treatment tolerance, and the patient's benefit–risk balance (55–57). From a clinical translational perspective, research on hypertension and frailty is gradually shifting from merely describing risk associations to an integrated management model centered on risk stratification, functional assessment, metabolic management, nutritional support, and exercise intervention. This also suggests that future studies should further clarify the optimal blood pressure control ranges for older patients with different degrees of frailty and explore the practical value of multicomponent interventions in improving cardiovascular outcomes, cognitive function, and physical function.

### 4.6 Citation landscape and clinically translatable evidence chain

Citation analyses further indicate that highly cited publications provide a coherent and clinically translatable evidence chain for hypertension–frailty research. The PURE cohort study, “Modifiable risk factors, cardiovascular disease, and mortality in 155,722 individuals from 21 high-income, middle-income, and low-income countries (PURE): a prospective cohort study” (1,156 citations), emphasized that hypertension remains a key driver among modifiable risk factors, with a particularly strong relationship with stroke risk. Importantly, PURE incorporated functional indicators such as grip strength and social determinants such as education into mortality risk frameworks, suggesting that management of older adults with hypertension should not be restricted to metabolic indicators or blood pressure values alone but should also consider functional reserve and vulnerability as determinants of long-term outcomes (22). Consistently, the SPRINT ≥75 years randomized trial, “Intensive vs. Standard Blood Pressure Control and Cardiovascular Disease Outcomes in Adults Aged ≥75 Years: A Randomized Clinical Trial,” demonstrated that targeting systolic blood pressure < 120 mmHg significantly reduced cardiovascular events and all-cause mortality; given the higher baseline risk in older adults, the absolute benefit (as reflected by NNT) is particularly notable. Exploratory analyses suggested that cardiovascular benefits of intensive treatment were also present among frail individuals or those with slow gait speed, implying that frailty does not necessarily indicate “insufficient benefit.” Retrospective evidence from HYVET similarly supports treatment benefits even in the most frail subgroup. A key trade-off is that intensive strategies may increase adverse events such as acute kidney injury, thereby requiring closer early follow-up, renal function monitoring, and medication optimization (24). Overall, these highly cited studies point toward a real-world-oriented framework: frailty should not be considered a reason to withhold antihypertensive treatment in older adults; rather, it should serve as a cornerstone for risk stratification and individualized target-setting. Incorporating grip strength, gait speed, and body composition into routine assessments, and layering function-oriented interventions (e.g., exercise and nutritional support) on top of blood pressure control, may help reduce disability and adverse outcomes and ultimately improve long-term quality of life. Consistent with local citation results, studies by Jeff D. Williamson and Jane Warwick remain highly cited, indicating sustained attention in specific regions and a shift toward clinically translatable research on treatment optimization and geriatric health management.

### 4.7 Limitations and future directions

Several limitations should be acknowledged. First, as a bibliometric mapping study, our findings depend on database coverage and retrieval strategies; relevant publications may have been missed or underestimated due to keyword selection, indexing delays, language bias, and differences in database scope—particularly for regional journals, non-English literature, and studies using heterogeneous terminology for frailty/functional decline. Second, citation metrics (e.g., total citations, local citations, and citation bursts) reflect academic influence and knowledge diffusion pathways rather than true clinical effect sizes or evidence quality. Highly cited publications may be influenced by earlier publication date, journal prestige, study type (e.g., large cohort studies and randomized trials tend to accrue more citations), and “hot-topic” effects, potentially amplifying certain themes. Third, although we used highly cited studies (e.g., PURE, SPRINT, HYVET) to interpret clinical implications, differences in inclusion/exclusion criteria and contexts should be considered. For example, SPRINT mainly enrolled ambulatory community-dwelling older adults and did not include high-complexity subgroups such as nursing-home residents, individuals with diabetes, or those with prior stroke; PURE and HYVET also differ in exposure measurement, outcome definitions, and healthcare-resource settings. Therefore, our synthesis regarding benefits of intensive blood pressure lowering in frail populations reflects consistency of evidence signals and should be further validated using real-world data and more representative high-risk subgroups. Fourth, keyword networks and clustering results are sensitive to analytic parameters (e.g., minimum occurrence thresholds, pruning strategies, and clustering algorithms), and different software or settings may yield variations in network structure and cluster labeling. Accordingly, this study is best interpreted as a macro-level summary of knowledge structure and research trends, providing hypotheses and directions for future refined clinical research, rather than as evidence to define specific clinical thresholds or replace guideline-grade evidence.

Future work may advance the field in several directions. First, at the bibliometric level, integrating additional databases and multilingual literature and refining search vocabularies (including terms such as frailty index, physical function, sarcopenia, and gait speed) may improve coverage; parameter sensitivity analyses and cross-software validation could enhance robustness of hotspot identification and thematic evolution conclusions. Second, at the clinical evidence level, more studies should include highly vulnerable and multimorbid subgroups (e.g., nursing-home residents, patients with diabetes or prior stroke, and individuals with markedly slow gait speed) to delineate benefit–risk boundaries of intensive blood pressure control across frailty strata and to emphasize functional outcomes relevant to older adults (falls, disability, hospitalization, and quality of life), rather than cardiovascular events alone. Third, intervention strategies should increasingly adopt integrated models of “blood pressure control + function-oriented management,” combining exercise, nutritional support, medication optimization, and monitoring, and should test feasibility, adherence, and cost-effectiveness in real-world settings. Overall, as the importance of comorbidity mechanisms and functional outcomes becomes increasingly recognized, future research is expected to move from risk identification toward stratified management and precision interventions, improving independence and long-term quality of life while reducing cardiovascular events in older populations.

## 5 Conclusion

Based on publications retrieved from WoSCC, Scopus, and PubMed, this study systematically mapped the knowledge landscape and evolutionary patterns of research at the intersection of hypertension and frailty. Overall, the literature has expanded rapidly since 2000 and accelerated markedly after 2015, indicating that hypertension–frailty comorbidity has emerged as a major focus in geriatric health management. At the country and institutional levels, the United States, China, and Italy were the leading contributors, and international collaboration networks have become increasingly interconnected, with top academic institutions playing a pivotal role in advancing the field. Keyword co-occurrence, clustering, and citation burst analyses revealed a relatively clear thematic structure. On the one hand, research has concentrated on hypertension management and downstream clinical outcomes (e.g., hospitalization, mortality, and complication risks). On the other hand, growing emphasis has been placed on frailty assessment tools (e.g., the *frailty index*) and functional indicators—including body composition, grip strength, and gait speed—as clinically meaningful instruments for risk stratification. Meanwhile, the rising prominence of themes such as “physical activity” and “successful aging” suggests a shift in research priorities from blood-pressure control alone toward broader strategies aimed at enhancing functional reserve and promoting healthy aging. Citation and local citation analyses further indicated that influential studies, including PURE, SPRINT, and HYVET, collectively support a clinically translatable message: in older adults with hypertension, frailty should not be viewed as a rationale to withhold treatment, but rather as a key determinant for individualized target setting, enhanced monitoring, and integrated interventions. Incorporating functional measures into routine assessment and layering function-oriented strategies—such as exercise, nutritional support, and medication optimization—may better reduce disability and adverse outcomes, ultimately yielding longer-term, real-world gains in quality of life. In summary, this bibliometric study not only delineates the global output patterns and knowledge structure of hypertension–frailty research, but also provides direction for future work. Further efforts are needed to refine frailty stratification and function-centered outcome evaluation, expand evidence coverage in highly vulnerable and multimorbid subgroups, and validate the effectiveness and feasibility of integrated “blood pressure control + function-oriented management” models in real-world settings, thereby informing precision interventions and healthy aging strategies in older populations.

## Data Availability

The data analyzed in this study is subject to the following licenses/restrictions: The datasets analyzed in this study were derived from the Web of Science Core Collection, Scopus, and PubMed, which are subscription-based databases. Licensing agreements and copyright restrictions prohibit the redistribution of raw downloaded records. The curated dataset used for analysis is not publicly available due to these restrictions and is not included in the manuscript or Supplementary material. However, the search strategies and inclusion criteria are fully described to ensure reproducibility. Requests to access these datasets should be directed to Shiyang Yu, 13558691884@163.com.
