# Supplementary material for: Hypertension and frailty in older adults: a bibliometric analysis and knowledge mapping based on Web of Science, Scopus, and PubMed (1973–2025)
Source: Front Med (Lausanne). 2026 Jun 18;13:1818161. doi: 10.3389/fmed.2026.1818161 (PMC13322900; doi:10.3389/fmed.2026.1818161)
Supplement: Supplementary file 1 [file Data_Sheet_1.docx]

**Supplementary File**

**Supplementary Table S1. Sensitivity analysis of the search strategy using broader frailty-related terms**

| **Database** | **Main search records** | **Sensitivity search records** | **Additional records** | **Increase (%)** | **Interpretation** |
| --- | --- | --- | --- | --- | --- |
| Web of Science Core Collection | 1,853 | 8,901 | 7,048 | 380.4% | Expanded terms markedly increased retrieval volume. |
| Scopus | 5,210 | 23,913 | 18,703 | 359.0% | Broader terms substantially reduced specificity. |
| PubMed | 1,615 | 7,720 | 6,105 | 378.0% | Broader terms introduced many potentially off-topic records. |
| **Total** | **8,678** | **40,534** | **31,856** | **367.1%** | **Expanded strategy greatly increased retrieval volume.** |

Abbreviations: WoSCC, Web of Science Core Collection.

Main search strategy: hypertension-related terms AND frail* / frailty. Additional records = sensitivity search records − main search records. Increase (%) = additional records / main search records × 100%.

Interpretation: the broader search terms substantially increased retrieval volume, suggesting that terms such as sarcopenia, functional decline, disability, or physical function decline may reduce specificity and introduce topic contamination if included in the main search strategy.

**Supplementary Table S2 Data integration, deduplication, and document-type standardization across databases**

| **Step** | **Number of records** | **Description** |
| --- | --- | --- |
| Records identified from Web of Science Core Collection | 1,853 | English articles and reviews retrieved from WoSCC |
| Records identified from Scopus | 5,210 | English articles and reviews retrieved from Scopus |
| Records identified from PubMed | 1,615 | English records retrieved from PubMed before document-type standardization |
| Total records before merging | 8,678 | Sum of records from the three databases |
| Records removed during deduplication and document-type cleaning | 3,724 | Duplicate records and non-target document types removed during preprocessing |
| Final records included in bibliometric analysis | 4,954 | Final cleaned dataset including articles and reviews |

**Note.** The 3,724 records removed during preprocessing included duplicate records and non-target document types; therefore, this number was not interpreted as duplicate records alone. Duplicate records were removed after standardizing DOI and title fields. For records with missing or inconsistent DOI information, title, publication year, and source information were additionally checked. PubMed records were standardized according to Publication Type to retain journal articles and reviews.

**Supplementary Table S3. Stability analysis of CiteSpace citation burst detection under different time-slicing parameters**

| **Burst item / theme** | **Representative burst keywords** | **1-year slice** | **2-year slice** | **3-year slice** | **Stability interpretation** |
| --- | --- | --- | --- | --- | --- |
| Antihypertensive treatment and therapy | antihypertensive treatment; therapy; active treatment; task-force | Detected | Detected | Detected | Stable |
| Blood pressure and hypertension management | hypotension; systolic blood pressure; isolated systolic hypertension; hypertension management | Detected | Detected | Detected | Stable |
| Older adults and aging population | older-adults; elderly/older-patient related terms | Detected | Detected | Detected | Stable |
| Clinical trial and double-blind evidence | trial; double-blind; double blind | Detected | Detected | Detected | Stable |
| Physical performance and functional status | performance; physical performance; activities of daily living | Detected | Detected | Detected | Stable |
| Frailty assessment | modified frailty index; frailty-related assessment terms | Detected | Detected | Detected | Stable |
| Cardiovascular outcomes and complications | heart-failure; complications; cardiovascular-related terms | Detected | Detected | Detected | Stable |
| Clinical characteristics and phenotype | clinical characteristics; phenotype | Detected | Detected | Detected | Stable |
| Nutrition-related topics | nutrition; malnutrition | Detected | Detected | Detected | Stable |
| Mechanisms and dysfunction | mechanisms; dysfunction | Detected | Detected | Detected | Generally stable |
| Endothelial dysfunction | endothelial dysfunction | Detected | Not detected | Detected | Partly stable / parameter-sensitive |
| Sex differences | sex differences | Detected | Not detected | Not detected | Less stable / parameter-sensitive |

Summary: The principal burst themes were largely preserved when the time-slicing parameter was changed from 1 year to 2 or 3 years, supporting the robustness of the citation burst results. Minor differences were mainly observed for narrower or more recent topics, such as endothelial dysfunction and sex differences.

**Supplementary Table S4. Sensitivity analysis of VOSviewer keyword co-occurrence networks under different occurrence thresholds**

| **Minimum keyword occurrence threshold** | **Number of keywords retained** | **Network density / readability** | **Main themes identified** | **Interpretation** |
| --- | --- | --- | --- | --- |
| 5 | 520 keywords; 8 clusters | Too dense | Hypertension; frailty; older adults; cardiovascular risk; physical function; cognitive function; outcomes; intervention management | High topic coverage, but the network was visually dense and contained more low-frequency terms, which reduced readability and interpretability. |
| 10 | 250 keywords; 7 clusters | Balanced | Hypertension; frailty; older adults; cardiovascular risk; cognitive function; physical function; intervention management; complications and outcomes | Selected for the main analysis because it retained the major thematic structure while reducing network noise and improving visualization clarity. |
| 15 | 174 keywords; 5 clusters | More simplified | Main themes were largely retained, including hypertension, frailty, older adults, cardiovascular risk, cognitive/physical function, and outcomes; however, some medium-frequency topics were reduced. | Higher specificity and clearer visualization, but lower topic coverage compared with the threshold of 10. |

**Note.** The number of retained keywords and clusters was obtained from VOSviewer using minimum keyword occurrence thresholds of 5, 10, and 15, respectively. Across the three thresholds, the core themes remained broadly consistent. A threshold of 10 was selected for the main analysis because it provided a balance between topic coverage and network readability.

**Supplementary Table S5A. Parameters used for institutional co-authorship analysis in VOSviewer**

| **Parameter** | **Setting** |
| --- | --- |
| Type of analysis | Co-authorship |
| Unit of analysis | Organizations |
| Counting method | Full counting |
| Handling of documents with many organizations | Documents co-authored by more than 25 organizations were ignored |
| Minimum number of documents per organization | 10 |
| Minimum number of citations per organization | 0 |
| Total organizations identified | 3,118 |
| Organizations meeting the threshold | 88 |

**Supplementary Table S5B. Top institutional contributors standardized by the final analytical dataset**

| **Rank** | **Institution** | **Publications, n** | **Standardized share (%)** | **Cumulative n** | **Cumulative share (%)** |
| --- | --- | --- | --- | --- | --- |
| 1 | Harvard University | 158 | 4.24 | 158 | 4.24 |
| 2 | Harvard Medical School | 155 | 4.16 | 313 | 8.40 |
| 3 | University of California System | 127 | 3.41 | 440 | 11.82 |
| 4 | Harvard University Medical Affiliates | 107 | 2.87 | 547 | 14.69 |
| 5 | Mayo Clinic | 98 | 2.63 | 645 | 17.32 |
| 6 | Johns Hopkins University | 97 | 2.60 | 742 | 19.92 |
| 7 | University of Toronto | 95 | 2.55 | 837 | 22.48 |
| 8 | Monash University | 94 | 2.52 | 931 | 25.00 |
| 9 | Graduate School of Medicine | 93 | 2.50 | 1024 | 27.50 |
| 10 | National Taiwan University Hospital | 84 | 2.26 | 1108 | 29.75 |

**Note.** Standardized share was calculated as the number of publications from each institution divided by the final analytical dataset (N = 3,724) and multiplied by 100. Full counting was used; therefore, one publication with multiple institutional addresses could contribute to more than one institution. The percentages should be interpreted as standardized contribution indicators rather than mutually exclusive proportions. Institutional names were harmonized during preprocessing when clear spelling variants or database-indexing variants referred to the same organization; ambiguous names were retained as indexed to avoid erroneous merging.

**Supplementary Table S6: Top 10 Most Cited Articles on Hypertension and Frailty: Local Citation Overview**

| **Number** | **First author** | **Article name** | **Journal name** | **Year** | **Local citations** |
| --- | --- | --- | --- | --- | --- |
| 1 | Jeff D Williamson | Intensive vs Standard Blood Pressure Control and Cardiovascular Disease Outcomes in Adults Aged ≥75 Years: A Randomized Clinical Trial | JAMA | 2016 | 142 |
| 2 | Jane Warwick | No evidence that frailty modifies the positive impact of antihypertensive treatment in very elderly people: an investigation of the impact of frailty upon treatment effect in the HYpertension in the Very Elderly Trial (HYVET) study, a double-blind, placebo-controlled study of antihypertensives in people with hypertension aged 80 and over | BMC Med | 2015 | 92 |
| 3 | Michelle C Odden | Rethinking the association of high blood pressure with mortality in elderly adults: the impact of frailty | Arch Intern Med | 2012 | 88 |
| 4 | Athanase Benetos | Treatment With Multiple Blood Pressure Medications, Achieved Blood Pressure, and Mortality in Older Nursing Home Residents: The PARTAGE Study | JAMA Intern Med | 2015 | 73 |
| 5 | Athanase Benetos | Hypertension Management in Older and Frail Older Patients | Circ Res | 2019 | 69 |
| 6 | Nicholas M Pajewski | Characterizing Frailty Status in the Systolic Blood Pressure Intervention Trial | J Gerontol A Biol Sci Med Sci | 2016 | 52 |
| 7 | Davide L Vetrano | Hypertension and frailty: a systematic review and meta-analysis | BMJ Open | 2018 | 51 |
| 8 | Athanase Benetos | An Expert Opinion From the European Society of Hypertension-European Union Geriatric Medicine Society Working Group on the Management of Hypertension in Very Old, Frail Subjects | Hypertension | 2016 | 43 |
| 9 | Rathi Ravindrarajah | Systolic Blood Pressure Trajectory, Frailty, and All-Cause Mortality >80 Years of Age: Cohort Study Using Electronic Health Records | Circulation | 2017 | 42 |
| 10 | Ivan Aprahamian | Hypertension and frailty in older adults | J Clin Hypertens (Greenwich) | 2018 | 41 |


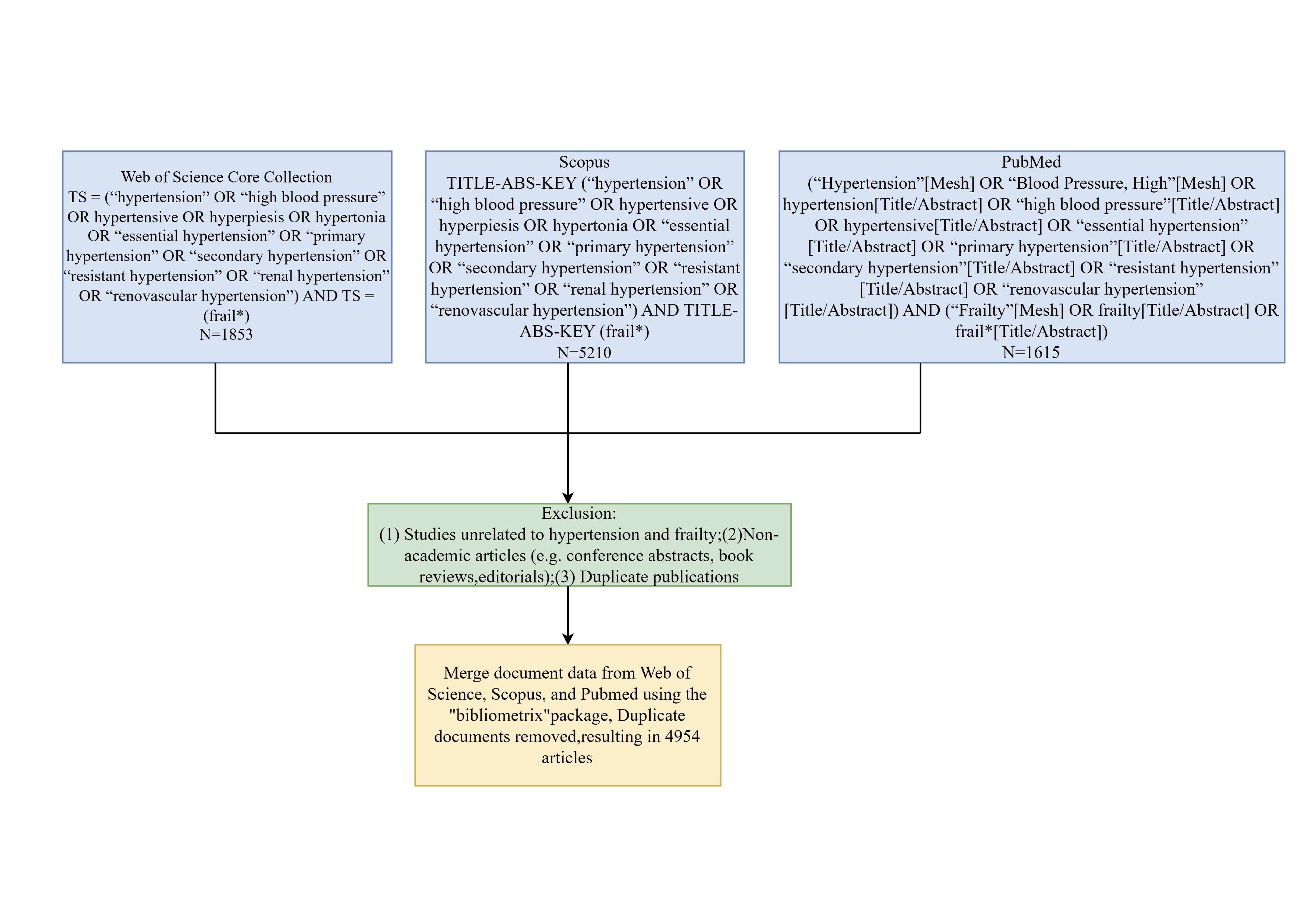


Supplementary Figure S1. Literature selection process for hypertension and frailty research
